# Endocrine Damper? Flame Retardants Linked to Male Hormone, Sperm Count Changes

**DOI:** 10.1289/ehp.118-a130b

**Published:** 2010-03

**Authors:** Kellyn S. Betts

**Affiliations:** **Kellyn S. Betts** has written about environmental contaminants, hazards, and technology for solving environmental problems for publications including *EHP* and *Environmental Science & Technology* for more than a dozen years

Additive flame retardants such as tris(1,3-dichloro-2-propyl) phosphate (TDCPP) and triphenyl phosphate (TPP), which are not chemically bonded to the products they are intended to protect, may escape into indoor environments such as homes, offices, and car interiors. A new study shows that men living in homes with higher amounts of TCDPP and TPP in their house dust had reduced sperm counts and altered levels of hormones related to fertility and thyroid function **[*EHP*** **118:318–323; Meeker and Stapleton]**. Because the research to date suggests both compounds are ubiquitous in U.S. homes, the study points to a pressing need for further investigation into the sources and levels of day-to-day exposure to the compounds as well as their potential health effects.

TDCPP has long been the main flame retardant used in automotive foam cushioning, while TPP has been used for decades in a wide variety of applications, including furniture foam. Since polybrominated diphenyl ether (PBDE) flame retardants were banned in Europe and discontinued in the United States in 2004, the use of alternative flame retardants such as TDCPP and TPP has been on the rise. Indoor dust is known to be an important source of exposure to PBDEs (which also are additive compounds), and the authors suspect this could also be true for other flame retardants.

In the current study, TDCPP was found in 96% and TPP in 98% of the house dust samples. As has been reported for other flame retardants found in house dust, the concentrations of the flame retardants in the samples varied markedly, with ranges of < 107–56,090 ng/g for TDCPP and < 173–1,798,100 ng/g for TPP. The concentrations of TDCPP in the men’s homes were comparable to those of PBDEs, whereas the levels of TPP were considerably higher.

Because the study participants were part of a larger project involving men recruited from a Boston infertility clinic, the authors had access to information about the men’s reproductive and thyroid hormone levels as well as their semen quality. They estimated associations for an interquartile range (IQR) increase in the level of each chemical measured in the dust samples, adjusting for potential confounders such as age and body mass. IQR analyses reflect the difference between the concentrations at the highest and lowest ends of the middle 50% of exposures.

This analysis revealed that each IQR TPP increase in the homes was associated with a 19% decrease in sperm concentrations and a 10% increase in prolactin levels. Increased prolactin can be a marker of decreased dopamine activity and also may be associated with erectile dysfunction. The authors also found that each IQR increase in TDCPP in the homes was associated with a 17% increase in prolactin and a 3% decline in free levels of the thyroid hormone thyroxine.

The findings mirror the limited toxicology data available on the study’s end points. They are also consistent with findings on other organophosphate compounds such as the urinary metabolite of the insecticide chlorpyrifos [*EHP* 112:1665–1670; Meeker et al.]. The authors hope to follow up by exploring human exposure pathways for these flame retardant chemicals and by reassessing these relationships with markers of endocrine function among a greater number of men from the larger ongoing study.

